# Insights into Personalised Medicine in Bronchiectasis

**DOI:** 10.3390/jpm13010133

**Published:** 2023-01-10

**Authors:** Clementine S. Fraser, Ricardo J. José

**Affiliations:** Host Defence, Respiratory Medicine, Royal Brompton Hospital, 37 Fulham Road, Chelsea, London SW3 6NP, UK

**Keywords:** bronchiectasis, endotypes, phenotypes, personalised medicine

## Abstract

Bronchiectasis is a heterogenous disease with multiple aetiologies resulting in inflammation and dilatation of the airways with associated mucus production and chronic respiratory infection. The condition is being recognised ever more frequently as the availability of computed tomography increases. It is associated with significant morbidity and healthcare-related costs. With new understanding of the disease process, varying endotypes, identification of underlying causes and treatable traits, the management of bronchiectasis can be increasingly personalised.

## 1. Introduction

Bronchiectasis is diagnosed radiologically and is characterised by irreversible dilatation of the bronchial tree. Clinically, its primary features are cough associated with excess mucus production, inflammation and thickening of the airways resulting in poor ability to clear sputum.

Many aspects of bronchiectasis, including its prevalence, underlying aetiology and presentation vary in different parts of the world [1,2,3].

**Figure 1:**
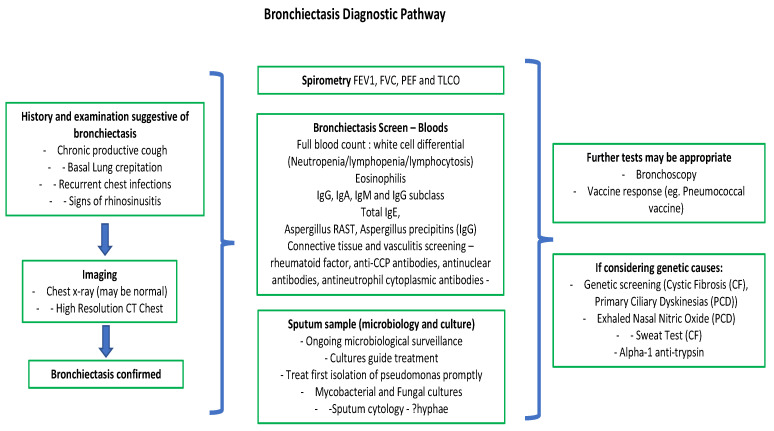
Bronchiectasis diagnostic flowchart.

It is increasing in prevalence both in the UK, Europe and across the globe [4,5,6]. Bronchiectasis has historically been underdiagnosed and long delays from the onset of symptoms to diagnosis of bronchiectasis have consistently been highlighted [7]. A typical diagnostic pathway can be seen in Figure 1.

The pathophysiology of bronchiectasis is underpinned by a vicious cycle consisting of airway infection, inflammation, impaired mucociliary clearance and structural damage causing progression of the disease. A widely recognised hallmark of bronchiectasis is its heterogeneity. Patients can present with varying severity of mucociliary dysfunction, a wide range of different microbiology and different patterns of inflammation and infection. Bronchiectasis, especially as it progresses, is associated with poorly reversible airflow obstruction, and often there is overlap with other airway centred diseases, notably chronic obstructive airways disease (COPD), asthma and allergic bronchopulmonary aspergillosis (ABPA).

An increasing focus has been made on the underlying molecular drivers and pathophysiological pathways (endotypes) of bronchiectasis as these can vary dramatically. Focusing on clinical phenotypes, microbiology, inflammatory endotypes, and condition overlap will allow us to provide an increasingly patient specific personalised therapy.

A rather rough aetiological classification distinguishes two main groups of bronchiectasis: cystic fibrosis (CF) bronchiectasis and non-CF bronchiectasis. The latter recognises a broad spectrum of causes and associations. The identification of CF bronchiectasis as its own entity with discrete pathological mechanisms has led to the identification of varying underlying genetic mutations, which has, in turn, spawned a new dawn of therapeutics in the form of CFTR modulators. A similar approach to other genetically identified causes of bronchiectasis may indeed lead to comparable therapeutic advances.

## 2. Phenotypes

The phenotype is the discernible characteristics of an organism resulting from the interaction between genetic and environmental factors. In bronchiectasis, the phenotype differs in clinical manifestations of the disease. For example, low vs. high mucus secretion, infrequent vs. frequent exacerbators, or by associated aetiologies or comorbidities.

### 2.1. Clinical Phenotype by Radiology

Radiology is key in the diagnosis of bronchiectasis. The bronchiectasis definition itself is underpinned by the permanent dilatation of the bronchial lumen. The advent of easily accessible CT scanning allows for rapid and non-invasive diagnosis. Radiological criteria include broncho–arterial ratio >1, lack of bronchial tapering, visualisation of peripheral bronchi within 1 cm of the costal pleura in contact with the mediastinal pleura, as well as indirect signs: peribronchial thickening, mucus plugging, mosaic pattern, centrilobular nodules, tree-in-bud nodules, focal areas of air trapping, atelectasis and consolidation [8].

**Figure 2:**
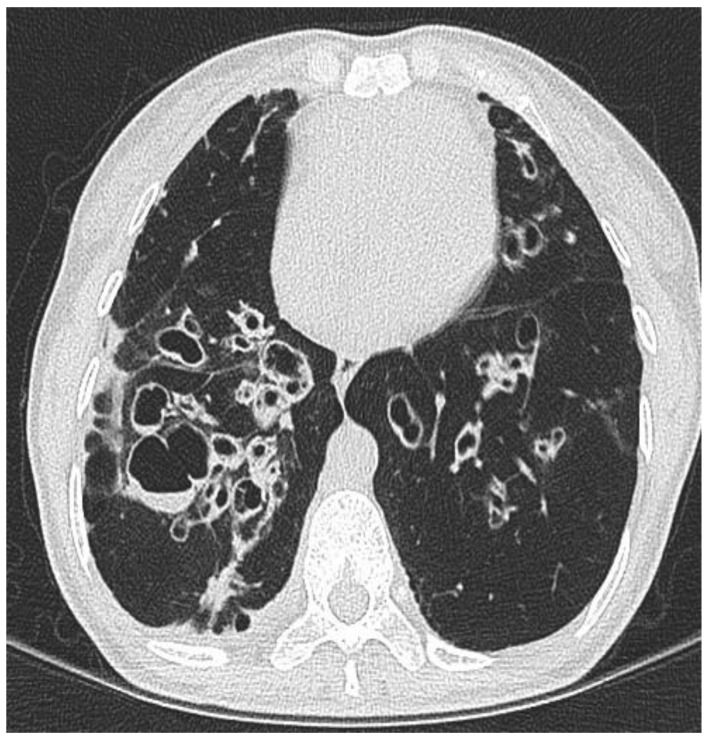
Computed tomography demonstrating cystic bronchiectasis in a patient with idiopathic bronchiectasis.

Radiological macroscopic morphology can be classified into three main types: the commonest is cylindrical bronchiectasis—where bronchi have a uniform calibre and do not taper (associated with the tram track and signet ring sign), varicose bronchiectasis—where bronchi are dilated and constricted in an irregular pattern, and cystic bronchiectasis—the most severe form where bronchi dilate to look cyst-like (Figure 2). The relative distribution of radiological bronchiectatic changes have been reported as: cylindrical 47%, Varicose 9.9%, cystic 45% and multiple types 24.3% [9]. Radiology can be highly influenced by underlying aetiology; CF-related bronchiectasis is often multilobar with an upper lobe predominance, ABPA tends to be seen in the upper and middle lobes, while bronchiectasis associated with connective tissue disease tends to be seen in the lower lobes. Recurrent infection and aspiration are associated with lower lobe disease, with the latter having a predilection for the right lower lobe [10].

### 2.2. Clinical Phenotype by Underlying Aetiology

Rough initial aetiological classification distinguishes two main groups of bronchiectasis: cystic fibrosis (CF) bronchiectasis and non-CF bronchiectasis. The latter encompasses a broad spectrum of causes and associations (Table 1). It is widely accepted that different causes of bronchiectasis may have diverse molecular pathways involved in their disease processes and therefore have potentially unique targets for treatments. Studies linking the underlying aetiology of bronchiectasis with outcomes have generally been small and have grouped aetiologies into post-infective bronchiectasis, non-post-infective bronchiectasis and idiopathic bronchiectasis [11]. Such broad grouping of aetiologies is not sufficient to identify unique phenotypes that may have specific associated endotypes or treatable traits. The problem with using aetiology to guide targeted intervention is that despite extensive assessment, an underlying cause for bronchiectasis may not be found, rendering the diagnosis-idiopathic bronchiectasis. There are large discrepancies in the proportion of patients reported to be without a known aetiology, depending on the cohort being investigated [12,13].

#### 2.2.1. Immunodeficiency Associated Bronchiectasis

In patients with antibody deficiency, immunoglobulin replacement therapy is often given to correct the humoral defect and reduce sinopulmonary infections. Once immunoglobulin replacement therapy is given, the disease trajectory for most people is in keeping with those with other causes of bronchiectasis. If immunodeficiency is not corrected, then a more severe disease progression is evident. It must be remembered, however, that despite receiving immunoglobulin replacement, bronchiectasis may still develop and progress with associated lung function decline [14].

#### 2.2.2. Primary Ciliary Dyskinesia Associated Bronchiectasis

Bronchiectasis associated with primary ciliary dyskinesia (PCD) is a rare ciliopathy characterized by recurrent upper and lower respiratory tract infections and may run a different course to other underlying pathological causes. It predominantly affects the respiratory tract ciliated epithelium. Generally, PCD is an autosomal recessive disorder however other modes of inheritance are also described (x-chromosomal [15] and autosomal dominant [16,17]). The underlying cilia may be drastically decreased in number but retain normal function or be defective in their utility. A large proportion (57% males and 48% females) of patients with PCD have situs inversus due to the cilia’s role during embryogenesis [18]. In addition, sperm cell flagella and the cilia lining the fallopian tubes and vasa deferens can be affected and infertility can occur. Clinical phenotype is influenced by mutation, for example, *CCNO* mutations result in severe, rapidly progressive lung damage, and are more frequently associated with hydrocephalus and infertility [19].

### 2.3. Clinical Phenotype and Overlap with Other Respiratory Conditions

It is important to recognize that the majority of bronchiectasis patients will have one or more overlapping respiratory pathologies, necessitating an individualised treatment approach. Many patients will have elements of other airway-centred diseases such as COPD and asthma. While each condition has its distinct pathophysiology, they are common characteristics: the presence of airway inflammation, airflow obstruction, airway remodelling and mucociliary dysfunction.

#### 2.3.1. COPD

The prevalence of bronchiectasis is high in COPD cohorts, especially those with severe disease. The CanCOLD study included healthy adults, smokers with normal lung function and patients with COPD and found a prevalence of bronchiectasis of 19.9% in both healthy individuals and smokers without COPD. This prevalence increased to 35% in severe COPD [20].

Having COPD and bronchiectasis together confers an increased mortality risk [21]. A UK-based study showed a 5-year mortality of 55%, compared with 20% in those with bronchiectasis without COPD [22].

#### 2.3.2. Asthma

The coexistence of asthma with bronchiectasis has been observed in many patients. It has been shown to increase the risk of exacerbations nearly three-fold. It can be difficult to clearly distinguish which of the two diseases has developed the acute exacerbation in asthma–bronchiectasis overlap patients [23,24].

#### 2.3.3. Allergic Bronchopulmonary Aspergillosis (ABPA)

Allergic bronchopulmonary aspergillosis (ABPA) occurs due to a hypersensitivity reaction to antigens of *Aspergillus fumigatus* following airway colonisation. ABPA is associated with the development of bronchiectasis in contrast to other *Aspergillus*-related diseases, which can establish themselves in patients with a background of bronchiectasis/architectural lung disease. The ABPA inflammatory phenotype has been associated with high levels of neutrophils and matrix metalloproteinases similar to those seen in certain bronchiectatic endotypes, suggesting overlapping pathophysiology [25]. Treatment for ABPA hinges on targeting fungal sensitisation with corticosteroids, reducing IgE-mediated inflammation with biological therapy and decreasing the airway fungal load with antifungals. Mucus tends to be viscous and mucolytics and airway clearance are essential to managing symptoms.

### 2.4. Clinical Phenotype by Severity and Prognosis

Classification of the severity of the disease can aid with treatment and follow-up decisions as well as patient planning. The most well-utilised severity scores are the Bronchiectasis Severity Index (BSI) [26] and FACED/E-FACED [27].

The Bronchiectasis Severity Index [BSI) has been validated in 1310 patients across 5 European bronchiectasis cohorts. It is scored by taking into account clinical, radiological and microbiological parameters. The total score is calculated by summing the scores for each variable and then classifying them into three severity classes: mild (0–4), moderate (5–8) and severe (>9) [26].

The FACED (Forced expiratory volume in 1 s (FEV1), Age, Chronic colonization, Extension, and Dyspnoea) score was developed and validated using a Spanish cohort, and showed it could accurately predict mortality [28]. It was recognised that while the FACED score has great prognostic capacity, it does not include the number or severity of exacerbations as a separate variable. The E-FACED was later developed, which has nine points of growing severity and has a superior prognostic capacity for exacerbations compared with FACED [27].

FACED and BSI have been compared and showed similar results with regard to severity and prognosis [29]. A comparative study of a pooled cohort of 1612 patients reported both scores had a good discriminatory predictive value for mortality. FACED consistently overestimated mortality in “severe” patients, with poor discrimination for hospital admissions, and did not consistently predict future risk of exacerbations [30].

It is well documented that all-cause mortality is higher in patients with bronchiectasis than in those without [5,26,31]. Mortality in bronchiectasis generally occurs from respiratory failure and respiratory infections. There are certain parameters that can predict the rate of decline and are associated with an increased risk of mortality. A longitudinal study reviewed mortality over a follow-up period of 13 years in 91 patients with radiologically confirmed bronchiectasis. Over this period, 27/91 (29.7%) patients died (more than double expected for the age cohort). The cause of death was bronchiectasis, respiratory infection or respiratory failure in 19/27 (70.4%). They reported that the independent impactors on mortality were age, SGRQ activity score, colonisation and TLC, RV/TLC and KCO lung function measurements [32,33].

Some studies have reported an increased risk of lung cancer in those patients with bronchiectasis. A recently published large Korean cohort study evaluated the incidence of lung cancer in 65,305 patients with bronchiectasis and in 3,793,117 without bronchiectasis, where they followed these patients over a 9-year period. They concluded that you were 28% more likely to have lung cancer if you had bronchiectasis independent of smoking compared with those without bronchiectasis. However, bronchiectasis did not increase the risk of lung cancer among participants with COPD [34]. It has been postulated that the underlying mechanism for this increased risk of lung cancer is chronic inflammation [35,36], much like the link already well described in COPD [37,38] and tuberculosis [39,40,41].

### 2.5. Clinical Phenotype by Microbiology

The presence of *P. aeruginosa* in bronchiectasis patients is repeatedly associated with increased mortality risk [22,32,33]. It is associated with an increased risk of exacerbation and hospitalisation [42], as well as more rapid lung function decline [43]. A study has shown that in those with chronic pseudomonas FEV_1_ declined on average 123 mL/year vs. 50 mls/year in those who were not *P. aeruginosa* colonised. A small retrospective study showed 80% success in the eradication of *P. aeruginosa* after first isolation, but this was short-lived as approximately half of patients cultured *P. aeruginosa* again at 6 months [44] In addition to pseudomonas, colonisation with *Haemophilus influenzae* has been shown to be associated with increased frequency of exacerbations but without the need for hospitalisation [45,46].

A significant proportion of bronchiectasis patients are colonised by non-tuberculous mycobacteria [47]. Mycobacterium avium complex (MAC) is the most common, including M. avium, M. intracellulare, and M. chimaera species [48,49]. Often these patients will have additional constitutional signs such as night sweats and weight loss. It has been reported that while isolation of NTM does not result in changes to clinical indices of disease severity (FEV1, body mass index, number of hospital admissions, and dyspnoea score), it does lead to worsened radiological features [50]. Often it has also been reported that those patients with NTM are more likely to have positive *Aspergillus* serology [51]. Certain radiological phenotypes are associated with specific NTM, for example. Lady Windermere syndrome refers to bronchiectasis in the right middle lobe or lingula segment due to Mycobacterium avium intracellulare infection, often present in elderly women who cough supress [52,53].

**Figure 3:**
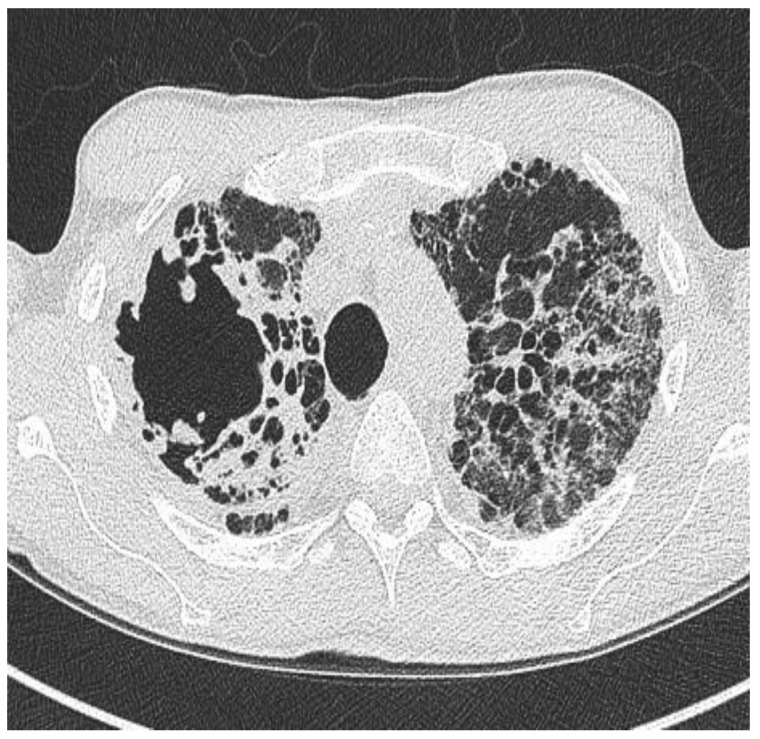
Chronic pulmonary aspergillosis in a patient with bronchiectasis mainly involving the upper lobes.

Aspergillus fumigatus may be cultured in the sputum of patients with bronchiectasis and particularly in patients with pre-existing cavities, such as those worth post-tuberculosis or COPD-related bronchiectasis, there is a risk of developing chronic pulmonary aspergillosis (Figure 3), with poor prognosis. ABPA has already been discussed.

## 3. Endotypes

An endotype is a subtype of a disease condition that is defined by a distinct pathophysiological mechanism.

The advent of technologies such as genomics, transcriptomics and proteinomics, coupled with the increasingly sophisticated bioinformatic and biostatistics programs, are allowing for novel biological analysis using big data to determine relevant disease biomarkers and their relationships. Use of these technologies is expanding due to growing understanding, more widespread implementation and reduced costs.

The continued characterisation of the main drivers of inflammation and infection in different bronchiectasis patient cohorts is key to finding targets for future therapy.

Highlighting these differing underlying mechanisms that may have a disparity of drug responses is a key area for further research.

### 3.1. Neutrophil Dysfunction

It is well-reported that neutrophil dysfunction is a common feature of several airway-centred diseases. Bronchiectasis is no exception. Understanding the mechanisms of neutrophilic inflammation is the key to identifying new targets for future drug development.

In stable bronchiectasis, blood neutrophils have been shown to display significantly prolonged viability, delayed apoptosis, increased CD62L shedding, upregulated CD11b expression, increased myeloperoxidase release, and impaired neutrophil phagocytosis and killing when compared with healthy volunteers. Neutrophil phagocytosis and killing is reported to be impaired at the start of an exacerbation but this improves following antibiotic treatment [54].

A study that analysed the serum and sputum of 245 bronchiectasis patients classified the biomarkers into three independent groups: eosinophilic and epithelial dominant (IL-5, IL-13 and Gro-α in sputum), systemic (GMCF, IL-6, VEGF, IL-10, and IL1β in serum) and airway neutrophilic inflammation (neutrophil extracellular traps, resistin, TNFα, CXCL-8, IL-10, MMP9 and elastase) [55].

Another study evaluated 14 inflammatory cytokines in soluble sputum and serum in 269 people with bronchiectasis. They mimicked inflammation by treating epithelial cell cultures with cytokines representing Th1 and Th2 inflammation (IL-8, IL-1β, IL-4 or IL-5) for 3 weeks, then assessed mucociliary clearance (MCC) and ciliary beating. They found MCC was decreased in the IL-8, IL-1β and IL-4 treated cultures. Ciliary beat amplitude and, therefore, efficacy was greatly reduced in the group treated with IL-8, IL-1β, IL-4 and IL-5. They concluded there is a similar effect on mucociliary clearance resulting from both Th1 and Th2 inflammatory cytokines [56].

Sputum neutrophil elastase and serum desmosine (a product of elastin breakdown) has long been a marker of disease severity and progression [57].

Many therapy target studies have focused on reducing this neutrophilic inflammation, however, this can have a knock-on effect of increasing the risk of infections through immunosuppression. Striking a balance is challenging and newer approaches include attempts to “reprogram” the neutrophils to reverse neutrophil dysfunction and the associated dysregulated inflammation. This so-called immunometabolic reprogramming is a process through which inflammation changes inflammatory cell behaviour by altering intracellular metabolic pathways. Studies show evidence that much of the neutrophil dysfunction observed in bronchiectasis is consistent with immunometabolic reprogramming [58].

### 3.2. Neutrophil Extracellular Traps (NETs)

The bronchiectatic airways contain factors such as resistin and high glucose levels. A recent study showed these promote AMP-activated protein kinase K inhibition, which in turn reduced ciliary beat frequency and increased neutrophil extracellular traps (NET) formation, therefore, highlighting AMPK activators as a therapeutic target in bronchiectasis [59].

NETs are large, webbed structures made up of DNA and microbial binding proteins; they can prevent microbial dissemination and actively degrade bacterial virulence factors [60]. However, proteases, antimicrobial proteins, DNA and histones released in this process can lead to excess inflammation causing tissue damage, impaired mucociliary clearance and impaired ability of innate cells to kill pathogens [61]. A number of studies have linked airway NET formation with greater disease severity, increased exacerbations and overall worse disease outcomes across the spectrum of airway diseases [62,63,64].

### 3.3. Eosinophilic/Type 2 Inflammatory Endotype

Although bronchiectasis is typically known as a neutrophilic disorder, eosinophilic subtypes are described. Eosinophils are well-established drivers of exacerbation in asthma and COPD, but over the past few years, they have been increasingly recognised as a key driver of inflammation in a specific subset of bronchiectasis patients.

A recent multicentre study that evaluated the blood and sputum eosinophils of over 1000 patient bronchiectasis exacerbations found that 20% of patients have an eosinophilic subtype. Blood eosinophil counts of ≥300 cells/μL were associated with a *Streptococcus*- and *Pseudomonas* bacterial microbiome. Blood eosinophils of <100 cells/μL were related to increased bronchiectasis severity and higher mortality rates. Higher blood eosinophil counts are associated with shortened time to exacerbation [65].

Another study examined a smaller cohort of patients (183) and found that while no association was observed between eosinophil counts and the frequency of exacerbations, those patients with serum eosinophil counts ≥300 cells/μL were more likely to have severe exacerbations [66].

A recent cross-sectional study explored how many patients with bronchiectasis but without asthma fell into the T2 inflammatory phenotype class. They defined this as the presence of either eosinophils blood count ≥300 cells·µL^−1^ or a fractionated exhaled nitrous oxide (FeNO) of ≥25 ppp. They found that 88/249 (35.3%) patients had either eosinophils ≥300 cells·µL^−1^ (53.4%) or FeNO ≥ 25 ppb (68.2%) 19 (9.4%) patients had both [67].

The identification of this subset of patients has led to interest in personalising treatment for this cohort to include steroid use and biologics more readily associated with asthma management.

In reality, patients often exhibit a combination of phenotypes. Over a third of patients (37.5%) in a bronchiectasis cohort exhibited a mixed neutrophilic and eosinophilic picture [68].

## 4. Treatments

Given the diverse nature of bronchiectasis, treatment is individually tailored to promote a pathophysiological, symptom-burden approach rather than a pan-syndrome method. Many patients have overlapping features with other respiratory diseases and addressing these as part of bronchiectasis treatment is paramount.

It is important different endotypes, phenotypes, and microbiology are reviewed, as well as overlapping conditions, as this that allows for increasing personalized treatments. Identification of the inflammatory drivers and exploring the associated ciliary phenotypes is paramount.

Treatment of bronchiectasis, broadly speaking, takes on three main categories: the improvement of mucociliary clearance, preventing and treating infection, and reducing associated inflammation.

### 4.1. Mucus Clearance

Improvement of mucociliary clearance relies on mucolytics, hyperosmolar agents and physiotherapy applying airway clearance techniques.

Reducing the production of mucus/improving sputum airway clearance is essential to disrupt the “vicious cycle” that is bronchiectasis disease progression.

Mucolytic agents target increased sputum viscosity to make sputum easier to clear. These include drugs such as hypertonic saline, N-acetylcysteine (NAC) [69], mannitol [70], erdosteine [71], recombinant human deoxyribonuclease (rhDNase) [72], and bromhexine [73], however, much of the evidence for these drugs is extrapolated from trials in cystic fibrosis associated bronchiectasis [74,75]. It must be noted, however, that in non-CF bronchiectasis patients, conflicting results have been shown, highlighting that exacerbations were more frequent and FEV1 decline greater in those patients who received rhDNase when compared with placebo [76]. It has been demonstrated that rhDNase reduces in vitro sputum transportability [77].

### 4.2. Antibiotics

One key foundation of bronchiectasis management is microbial surveillance. This is done through frequent sputum cultures, oropharyngeal swabs and/or bronchoscopy samples when required. Prompt identification and treatment of acute infective exacerbations with oral or intravenous antibiotics is paramount. However, we must be mindful of increasing antibiotic resistance.

Antibiotics are also used as prophylaxis in those with the tendency to frequently exacerbate. Those most commonly in use for prophylaxis are azithromycin and doxycycline. Long-term use can have side effects that require monitoring. For example, azithromycin can result in prolonged QT/cardiac arrythmias and ototoxicity/tinnitus, and doxycycline can cause gastrointestinal disturbance and increase skin photosensitivity. Some antibiotics can also be delivered in nebulised form.

### 4.3. Corticosteroids

The role of inhaled steroids in bronchiectasis has been shown to improve symptoms in stable disease [78] as well as reduce inflammatory markers in sputum and reduce sputum volume; they do not, however, reduce exacerbation frequency [79]. It has been postulated that blood eosinophils can help predict those for whom inhaled corticosteroids are more beneficial, with those displaying high T2 endotype deriving more benefit. [80,81,82]. Although consideration must be given to the potential increased risk of respiratory infection, non-tuberculous mycobacteria disease, adrenal suppression and deterioration in bone density.

The benefit of oral corticosteroids in acute or stable bronchiectasis has not been proven. It would be reasonable to assume that oral steroids may be helpful in certain individuals. For example, in those with COPD/asthma/ABPA overlap or those who display a more type 2 inflammatory picture. More large-scale studies are required to further evaluate their use in the context of different endotypes.

### 4.4. Surgery

Despite the aggressive conservative therapies above, there are cohorts of patients for which surgery is beneficial and appropriate; these are generally patients with unilateral, localised disease with persistent symptoms. For the most part, patients undergo lobectomies which have been shown to be safe with a low rate of complications [83,84,85].

The above constitutes the mainstay of therapy for bronchiectasis. Currently, there are limited therapies that target host inflammatory and epithelial cells. Identifying therapies that can modulate inflammation and mucociliary dysfunction is imperative to improve outcomes. Trials are underway and it is hoped new therapies that target these pathways will come to market soon.

## 5. New Therapies

Our increased understanding of the pathophysiology of bronchiectasis highlights the vast heterogeneity of the disease. Knowledge of the different biological mechanisms underpinning the disease process gives hope that the identification of certain traits will allow for tailored, individualised management plans.

### 5.1. Brensocatib

Brensocatib is an oral reversible inhibitor of dipeptidyl peptidase 1 (DPP1), an enzyme involved in the activation of neutrophil serine proteases. DPP1 inhibition prevents the activation of the neutrophil protease’s elastase, cathepsin-G and proteinase-3 in the bone marrow. The recently reported WILLOW trial showed a primary outcome that brensocatib significantly delayed time to exacerbation (134 days vs. 67 in placebo group), secondary outcomes showed reduced rates of exacerbations and significant drops in sputum neutrophil elastase levels. Brensocatib has now entered much larger, longer phase 3 trials (ASPEN) [86]. Brensocatib has received Breakthrough Therapy Designation from the US Food and Drug Administration (FDA) as well as Priority Medicines (PRIME) designation from the European Medicines Agency (EMA) for patients with bronchiectasis.

### 5.2. Biologics

For those patients who display an eosinophilic/type 2 endotype there is increasing interest in the effect biologic agents may have on their disease. Over the past 10 years, the use of biologics has revolutionised the treatment of asthma [87,88]; for example, a study reported treating five patients with severe eosinophilic asthma and bronchiectasis with mepolizumab or benralizumab. This significantly reduced the exacerbation rate with an effect that persists for up to 2 years [67]. Although this study is small, similar results have been replicated [89].

### 5.3. PCD-Specific Therapy

Identification of precise genotypes causing PCD has allowed for the principle mechanisms of cilia dysfunction to be characterised. Given this, there has been increased attention on gene therapy, focusing on both replacing or modifying the mutated gene sequences.

The *DNAI1* gene is responsible for approximately 10% of PCD cases [90] it encodes an important component of the outer dynein arm (ODA) of the cilia. Studies have used lentiviral vectors containing cDNA of *DNAI1* to incorporate its genetic material into the host genome; this has resulted in some cilia beating activity being restored [91]. Others have replicated similar findings carrying out classical gene therapy in mouse models with some restoration of ciliary function [92,93,94]. While gene therapy attempts to add correct copies of the gene into the genome of the cells, gene editing alters the genome just at the specific location of interest to correct the genetic sequence. Gene editing has been attempted in epithelial cell lines containing the *DNAH11* mutation. It has been shown that it is possible to replace the mutated sequence with a wild-type sequence in about 50% of cells. Recombination and normalisation of ciliary beating and pattern occurred in 33% and 29% of cells, respectively [95]. While technically possible, concerns remain around the safety of gene therapy/editing [92]. In the last few years, there has also been interest around the use of transcript/RNA therapies in the treatment of PCD [96]

With the advent of more sophisticated technological tools, the means to identify drug targets for PCD have become ever closer. A recent study highlighted a platform for higher throughput analyses of airway epithelia advocating new possibilities for drug evaluation and development in patients with PCD caused by nonsense mutations [97].

### 5.4. Phage Therapy

Phages (or bacteriophages) are viruses that are used specifically to target bacteria. Phage therapy involves using phages to treat bacterial infections. A body of research has been undertaken for difficult-to-treat bacteria.

Mycobacterium abscessus complex is well known as a highly resistant bacteria that can cause significant morbidity and mortality, especially in the immunocompromised host and those with underlying structural respiratory diseases such as bronchiectasis. It is very difficult to treat due to its intrinsic and acquired resistance. Phage therapy is, however, showing some promise in small-scale case reports. Recently, a 15-year-old patient with cystic fibrosis (homozygous for ΔF508) was diagnosed with disseminated *M. abscessus* subsp. *massiliense* infection post lung transplant. A 3-phage cocktail was produced and applied to a sternal wound and given intravenously. The patient was discharged from hospital 9 days later with the continuation of the phage cocktail.

Phage therapy has also been shown to be of use in pseudomonas; in vitro and in vivo studies have shown that phages can destroy biofilms and thus increase permeability to antibiotics [98,99]. In a CF zebrafish model infected with pseudomonas phage therapy has decreased lethality, bacterial burden and the pro-inflammatory response [100]. There is currently a US-based trial looking at the use of nebulised phage therapy in the CF cohort with pseudomonas; preliminary results are due next year [101].

## 6. Conclusions

Reassuringly, interest and research activity in bronchiectasis seems to be gathering momentum. Large patient datasets are being collated, and this coupled with the increasing availability of new technologies such as multiomics, whole genome sequencing, proteonimcs, metabolomics, single-cell RNA sequencing and lipidomics should allow for the identification of specific phenotypes and endotypes that can be therapeutically targeted. In combination with our increasing knowledge regarding pathogens themselves and their interactions with hosts, the next decade promises to be an exciting era.

## Figures and Tables

**Table 1:** Aetiologies of bronchiectasis.

Bronchiectasis Aetiologies	Examples
Idiopathic	
Post Infection	Mycobacterial infection. Bacterial pneumonia, e.g., Bordetella Pertussis (especially in childhood). Viral infections—measles, influenza, adenovirus (especially in childhood). Aspergillosis Swyer–James syndrome (secondary to childhood bronchiolitis obliterans).
Impaired immunity	Primary immunodeficiency disorder, e.g., common variable immunodeficiency (CVID), hypogammaglobulinaemia, chronic granulomatous disease, Waldenstrom macroglobulinemia. Acquired—e.g., 3e HIV, malignancy (haematological), chemotherapy, post-transplant.
Genetic/Congenital	Cystic fibrosis, primary ciliary dyskinesia, alpha-1-antitrypsin deficiency. Bronchial tree malformations (tracheomegaly (Mounier–Kuhn syndrome)), cartilage deficiency (Williams–Campbell syndrome), pulmonary sequestration, bronchial atresia.
Allergic/Autoimmune	ABPA, asthma, COPD. Connective tissue diseases—e.g., rheumatoid arthritis, systemic lupus erythematosus (SLE), Sjogren’s, relapsing polychondritis inflammatory bowel disease
Obstruction	Foreign body, malignancy, anatomical abnormality.
Inflammation	Aspiration, radiation induced, toxic inhalation (chlorine, ammonia, smoke).
Malignancy	Primary lung—bronchogenic carcinoma, bronchial carcinoid. Thymoma (via means of hypogammaglobulinaemia).
Chronic lung diseases	Granulomatous—sarcoidosis, interstitial pneumonias (often via means of traction bronchiectasis). Post lung transplant.
Other	Youngs disease. Yellow nail syndrome.

## Data Availability

Not applicable.
